# New technologies applied to neonatal transport

**DOI:** 10.1186/1824-7288-41-S1-A26

**Published:** 2015-09-24

**Authors:** Michele Panico

**Affiliations:** 1 Neonatal Intensive Care Unit and Neonatal Emergency Transport, A.O.R.N. Sant'Anna e San Sebastiano, Caserta, Italy

## 

As neonatal care in the tertiary setting advances, neonatal transport teams are challenged with incorporating the innovations into their work environment. Some advancements over the last years involve communication, respiratory management, hypothermia, newborn comfort.

## Communication

The communication gold standard is the implementation of advanced technologiesas establishment of areal-time telepresence clinical network that allows online collaboration between primary care physicians working in community hospitals and critical care transport teams on moving vehicles. These participants will be able to work in collaborationduring the evaluation, stabilization and transfer of critically ill newborns.

## Respiratory management

Many major respiratory treatments and the equipment required have been adapted for transport. There is evidence that new methods of non invasive ventilation support have significantly changed RDS management in preterm infants. Further perspectives for neonatal transport teams involve the assessment of NIV strategies. If the infant is less than 28 weeks, has an air leak, or has persistentpulmonaryhypertension, the team may elect to place the infant on high frequencyventilation. To date two modes of HFV has been studied in the care of infants: high frequency oscillatory ventilation (HFOV) and high frequency jet ventilation (HFJV). However, transport with the HFO is not a current option as it does not have external battery power. HFJV can be used for transport as it has an external battery. After ventilation is established, the team assesses the need for surfactant. The administration of surfactant prior to transport determine a significantly greater drop in oxygen requirement and appears to be very safe. Infants requiring high ventilator support, such as infants with PPH, may require conventional ventilation and inhalednitricoxideor more commonly iNO and HFV. Infants are usually started on iNO at 20 ppm, a dose that has been found very effective to achieve positive results.

## Hypothermia

Ideally all infants who are being considered for cooling should have a preliminary CFM recording prior to starting active cooling. A new system has been purchased to provide a portable means of undertaking a CFM assessment at referring sites for infants who are being considered for transfer for therapeutic cooling. In these infants the use of active cooling using servo-controlled cooling mattress during transfer achieves target temperature in a significantly shorter period and maintains better temperature stability during transfer.

## Newborn comfort

All babies showed higher levels of discomfort during transport. Discomfort is increased by mechanical ventilation or other invasive procedures. Analgo-sedation improve outcome of newborn during transport.
